# COVID-19 vaccination refusal among the anti-vaccinationists in a Chinese society: a critical medical anthropology study of the vaccination barriers

**DOI:** 10.3389/fpubh.2025.1495951

**Published:** 2025-03-11

**Authors:** Judy Yuen-Man Siu

**Affiliations:** ^1^Department of Applied Social Sciences, Faculty of Health and Social Sciences, The Hong Kong Polytechnic University, Kowloon, Hong Kong SAR, China; ^2^International Research Centre for the Advancement of Health Communication, The Hong Kong Polytechnic University, Kowloon, Hong Kong SAR, China; ^3^Research Centre for SHARP Vision, The Hong Kong Polytechnic University, Kowloon, Hong Kong SAR, China

**Keywords:** anti-vaccination, COVID-19, barrier, resistance, political economy, critical medical anthropology

## Abstract

**Introduction:**

This study investigated the reasons for COVID-19 vaccination refusal among some Hong Kong residents who were anti-vaccinationists, despite the implementation of a vaccine incentive policy called the Vaccine Pass. The health belief model and the theory of planned behavior have been widely employed to analyze the determinants of COVID-19 vaccination. However, these two theories focus on the micro individual factors, which do not provide a sufficiently comprehensive analysis.

**Study design:**

A qualitative descriptive approach with a critical medical anthropology framework.

**Methods:**

This study adopts a critical medical anthropology framework that provides a micro and macro analysis at four social levels. A qualitative approach with individual, semi-structured, in-depth interviews was conducted from September 2022 to March 2023 with 30 individuals aged 20–59 years who did not receive COVID-19 vaccination in Hong Kong. The participants were recruited through purposive sampling and snowball sampling. A thematic analysis of data was implemented.

**Results:**

The reasons for COVID-19 vaccination refusal involved intertwining relationships among factors in the four social levels of the critical medical anthropology framework. The participants’ doubts about the safety of COVID-19 vaccines at the individual level were interacting with: (1) their ethnocultural beliefs and the perceived profit-oriented nature of vaccine production and distribution at the macro-social level, (2) their interpretation of the inconsistent advice of medical doctors at the micro-social level, and (3) their distrust in the government’s vaccination policies at the intermediate-social level.

**Conclusion:**

The participants’ refusal of COVID-19 vaccines was correlated with perceived profit motives related to the vaccine, perceived conflict of interest of health-care providers, and the distrust of government.

## 1 Introduction

As of December 2024, the coronavirus disease 2019 (COVID-19) pandemic had resulted in more than 777 million confirmed cases and a death toll exceeding 6 million worldwide (1). COVID-19 vaccination is regarded as one of the most effective measures of preventing infection (2). As of January 2024, more than 13 billion COVID-19 vaccine doses had been administered worldwide (3), and around 67% of the world population has received a complete primary series of COVID-19 vaccine as of December 2023 (3). To encourage vaccination, the Hong Kong government implemented the Vaccine Pass policy on February 24, 2022 (4). The Vaccine Pass was an optional vaccination incentive policy that linked an individual’s personal access to certain public venues and services based on their COVID-19 vaccination status. This policy allowed those who had completed the target number of vaccine doses to enjoy a higher level of social access, such as dining in instead of ordering take-out and visiting schools and medical facilities; those who had not yet received the target number of vaccine dose were not allowed to enter these public venues and facilities (4). The Vaccine Pass was implemented in three stages, with the first stage beginning on February 24, 2022, and the final stage beginning on May 31, 2022 (4).

Statistics revealed that the vaccination rate increased considerably after the implementation of each stage of the Vaccine Pass (4). As of September 27, 2022, when the Vaccine Pass was fully implemented and enforced, 94.1% of the Hong Kong population had received one vaccine dose, 91.8% had received two doses, and 77.8% had received three doses (5). The vaccination rate had further increased as of March 2023, when the Vaccine Pass had ended, with 94.6% of the population having received one vaccine dose, 93.1% having received two vaccine doses, and 84% having received three vaccine doses; in addition, approximately 15.8% of the population had received additional fourth and fifth vaccine doses (5). Therefore, the Vaccine Pass strongly motivated the people of Hong Kong to receive a COVID-19 vaccine, which is consistent with the past international studies reporting that a mandatory vaccination policy or vaccination incentive policy is crucial to reach high vaccination coverage (6, 7). Governmental vaccination policies, thus, are an influential factor for increasing vaccination rates.

However, as of September 2022 and March 2023, approximately 5.9 and 5.4% of the Hong Kong population remains unvaccinated against COVID-19, respectively. Although this percentage is statistically insignificant, this subpopulation can still pose a potential threat to public health if future outbreaks of COVID-19 occur. The World Health Organization has listed anti-vaccination as one of the top 10 health threats for the world in 2019 (8). Therefore, investigating the reasons why this subpopulation refuses to be vaccinated, despite the largely successful vaccination incentive policy implemented by the Hong Kong government and in other western countries (5–7), is essential to inform the creation of a more effective and sensitive vaccine promotion strategy.

Studies have examined the barriers to vaccination against COVID-19 among various groups, such as older adults (9), ethnic minorities (10), healthcare providers (11), and parents (12). However, relevant studies have primarily focused on vaccine hesitancy, which is a continuum between delaying of acceptance or refusal of vaccines (13). The relevant literature of vaccine hesitancy can therefore include study samples who are not necessarily opposed to or refusing vaccination. Literature about the barriers of anti-vaccinationists to receiving COVID-19 vaccination is rare. This study fills the literature gap by examining individuals who are anti-vaccinationists for their reasons of refusing to be vaccinated against COVID-19.

### 1.1 Theoretical framework

The health belief model and the theory of planned behavior that mostly focused on personal and individual attributes (14) have been widely applied to analyze vaccination-related behavior, including the determinants of COVID-19 vaccination (15–22). These past studies commonly point to personal knowledge and attitude, risk perceptions, perceived behavioral control, and subjective norms as significant factors in affecting people’s willingness of COVID-19 vaccination. However, vaccine refusal is a complex, multifaceted construct that is rooted in the sociocultural structures of a society that guide people’s choice (23). Hence, health belief model and the theory of planned behavior would be limited in providing a sufficiently comprehensive analysis in vaccination decisions. Indeed, Limbu et al. (18) and Lin et al. (19) indicated that in addition to the factors concerned in the health belief model and the theory of planned behavior, socio-economic factors such as gender, education level, and income can also affect people’s willingness of vaccination. Besides, the manufacturing place of vaccine is also indicated as a significant factor for the people of Mainland China in considering COVID-19 vaccination, as foreign-made vaccines were shown to be related to the higher vaccine hesitancy among the Chinese-in-Mainland (19).

It is therefore important to consider social and cultural aspects in understanding people’s vaccination behavior. The SAGE Vaccine Hesitancy Working Group of the World Health Organization suggests vaccine hesitancy should be understood from a social-ecological focus, and it can include factors of complacency, convenience and confidence that cover contextual (i.e., historic, sociocultural, environmental, health system/institutional, economic or political factors), individual and group influences (i.e., personal perception of the vaccine, influences of the social/peer environment), and vaccine/vaccination specific issues (13). Brewer (24) further suggests The Increasing Vaccination Model in understanding people’s vaccination behavior. This model indicates vaccination uptake is a complex construct of different social and cultural forces, involving what people think and feel about the risk and confidence of vaccines, social processes involving social norms, social networks, and sense of altruism, and direct behavior change.

However, what can affect these social and cultural forces in vaccination determinants remains largely understudied. Indeed, past studies note that there are macro factors affecting these social and cultural forces, and anti-vaccination is viewed as civil disobedience and public distrust of scientific medicine and ‘new science’ (25). Following Porter’s and Porter’s argument that anti-vaccination is a reflection of anti-medicine and anti-science as the political and ideological backstage (25), this study therefore expands the dialogue of World Health Organization’s and Brewer’s frameworks on vaccine hesitancy by adopting critical medical anthropology (CMA) (26) as the theoretical framework in guiding a more comprehensive analysis in COVID-19 vaccination decision-making that involves both the micro and macro social aspects, as the CMA framework argues that the political economy of a society as a social-ecological environment is a key basis for health. According to CMA, the mutual interacting political and economic forces can affect human relationships, collective experiences, and social behaviors in health (26). Following this sense, vaccine refusal should be seen as a product originating from these intertwining political and economic forces. Albrecht (27) shows that people’s political views is a strong determinant for COVID-19 vaccination acceptance in the United States, as people’s anti-vaccination was related to their political opposition to the county governments. Canwat (28) further demonstrates the political economy behind the COVID-19 pandemic, which has affected public and private policy actors in vaccination policy and ultimately people’s vaccine acceptance in his case study of East Africa. The CMA framework is adopted in this study as it provides a systematic analysis of how the political economy of a society can affect anti-vaccinationists’ behaviors and perceptions through investigating factors at the individual, micro-social, intermediate-social, and macro-social levels (26). At the individual level, personal factors and social support networks influence a person’s health-related behaviors and perceptions (26). At the micro-social level, the interaction between a person and their healthcare providers influences the person’s health-related behavior (26). A person’s health-related behaviors and perceptions are also affected by institutional and government policies at the intermediate-social level and by ethnocultural-medical beliefs and capitalistic ideology at the macro-social level (26). This study employed the CMA framework to investigate the embedded political economy of vaccination behavior among the anti-vaccinationists to be vaccinated against COVID-19.

## 2 Methods

### 2.1 Design

This study adopted a qualitative descriptive research approach using individual semi-structured interviews for data collection. Qualitative research is widely used to understand patterns of health behaviors as well as people’s lived experiences and healthcare needs in order to design and inform appropriate health interventions (29, 30). This inquiry examines the “how” and “why” of healthcare decision making (31) and explains the phenomena by making sense of the complex social reality (32). A qualitative descriptive approach provides straightforward descriptions of participants’ perceptions and experiences (33). This approach recognizes the subjective nature of the problem, and the findings are presented to reflect or closely resemble the terminology used in a research question (34). It provides factual responses to how people feel and experience (35), and researchers can stay closer to the data and the words used by informants as this approach emphasizes a lower level of interpretation and inference from researchers (36). Qualitative descriptive approach matches with the anthropological approach adopted in this study as anthropological study mostly involves descriptive analysis that puts relationship of causality into contextualization in social context (37). A thematic analysis of data was implemented in accordance with the critical medical anthropology (CMA) framework.

### 2.2 Data collection

The author of the present study conducted individual, semi-structured, in-depth interviews with 30 anti-vaccinationists against COVID-19 vaccine (19 men and 11 women) in Hong Kong from September 2022 to March 2023. The sample size of 30 anti-vaccinationists in this study was referenced from a grounded theory qualitative study on the experience of 21 vaccine refusing parents from regional and urban locations in five states of Australia (38). Participants were recruited using purposive sampling, and they satisfied the following criteria: (1) age 20–59 years, (2) had never been vaccinated against COVID-19, (3) had indicated refusal of receiving COVID-19 vaccination, (4) were of Chinese ethnicity, and (5) were residents of and had been educated in Hong Kong. These criteria were formulated to ensure that the participants had a long exposure to Hong Kong society and its culture and they were anti-vaccinationists of COVID-19 vaccination, which facilitates the investigation of the vaccine refusal in relation to the social-ecological perspective according to the CMA framework.

The COVID-19 vaccination rate in Hong Kong was closely tied to the implementation of the Vaccine Pass. The Vaccine Pass was implemented in three stages; in the final stage, which began on May 31, 2022, only individuals who had received three vaccine doses were allowed to enter certain public facilities (4). The participants were recruited from September 2022 to March 2023. This timing enabled the recruitment of anti-vaccinationists of COVID-19 vaccination and hence the investigation of their vaccination refusal.

Those who refuse vaccination can be regarded as a hard-to-reach, hidden, and vulnerable population because this population is often invisible (39, 40). Therefore, the 30 participants in this study were recruited from different sites. In the first stage of participant recruitment, recruitment posters listing the sampling criteria were posted in public areas of a local university, and one participant was recruited through this channel. In the second stage of participant recruitment, 29 participants were purposively sampled in the community through a clinic assistant’s Facebook fan page in addition to snowball sampling, which involved recruiting participants referred by other participants. Using social media platforms such as Facebook in participant recruitment has become popular in sampling hidden populations (41). Studies involving hard-to-reach and vaccine-hesitant populations have supported the sampling of participants through Facebook and by using snowball sampling (42–45). The clinic assistant’s Facebook fan page is written in the local dialect of Hong Kong with more than 200,000 followers during the data collection period, which facilitated the purposive sampling of the participants according to the sampling criteria. With the consent of the owner of the Facebook fan page, an e-poster was posted onto this page for participant recruitment.

An interview question guide (Appendix A) with an inductive design was employed. The interview question guide was developed with reference to the literature on the barriers to receiving COVID-19 vaccination (15–22) and on the basis of fieldwork observations in Hong Kong. The interview questions were open-ended to allow the participants to flexibly share their experiences. The participants were interviewed individually by the author to ensure the consistency of interviews. The author has a training background in anthropology at the Bachelor’s and Master’s levels, and in medical anthropology at the research doctorate level (Ph.D). She did not know the participants before the interviews to ensure the interviews could be conducted with minimal bias. All the interviews were conducted in Cantonese, which is the native tongue of the researcher and the participants. Selected interview quotes used in reports were translated from Cantonese to English, and back translation was employed to ensure the accuracy of quotes.

Because the interviews were conducted with the vulnerable population, all 30 interviews were conducted online by using Zoom or WhatsApp video calls, as the use of virtual interviews in catering the needs of informants from marginalized population have been documented in past literature (46). The author interviewed the participants in a private room at her institution to protect the participants’ confidentiality. Each interview lasted between 50 min and 1.5 h, and the audio was recorded with the participant’s consent. Each participant was compensated with a supermarket cash coupon worth HK$200 (approximately US$25.64) by mail upon completion of the interview.

### 2.3 Data analysis

After each interview, the author documented the key points and her impressions and observations in an interview diary. This procedure involves quick and initial data analysis that can inform the later data collection process. The interviews were then transcribed verbatim. The interview data were subjected to thematic analysis following the procedure of Braun and Clarke (47). All transcripts were read and reread line by line, giving the author a comprehensive understanding of the entire experience of the participants (48). All statements or phrases that were relevant to the participants’ refusal to vaccination and those descriptions that were relevant to this phenomenon were identified, extracted, described, and translated into codes, which were then labeled and categorized. Overlapping codes were consolidated to form broader categories. Recurrent categories were highlighted, and were then consolidated to form themes after repeated examination and comparison. The data analysis process involved documenting codes, categories, themes, and supporting interview quotes derived from the data in a coding table (49). Specific concepts and categories were highlighted in the coding table to transform the interviews into meaningful symbols to enable a deeper understanding of the participants’ thoughts. The four social levels within CMA framework served as the backbone throughout the data coding and analysis procedure.

### 2.4 Ethical considerations

Ethical approval was obtained from the Human Subjects Ethics Subcommittee of The Hong Kong Polytechnic University before the study began (approval reference number: HSEARS20200924003). The participants were informed about the purpose of this study and granted informed consent prior to their interview. All interviews were conducted anonymously, and each participant was represented with a code to ensure confidentiality. All collected data were stored in password-protected computer files that were only accessible by the researcher.

### 2.5 Research rigor

The criteria developed by Lincoln and Guba were used to ensure the robustness of the study design and methods (50). Data saturation was achieved. No new themes or codes were emerged and informational redundancy was occurred since the 26th interview, and the data obtained from four additional interviews afterwards did not yield any new data to confirm data saturation (51). Member checking was implemented, and the participants were asked to review the transcripts and interview diaries of their interviews to ensure the accurate portrayal of their viewpoints (52). Quotes from interviews were included in the coding table to ensure that the codes, categories, and themes were grounded in the interview data (49). Cross-checking between the interview quotes, codes, categories, and themes was implemented throughout the analysis. As this study follows the CMA framework and requires the researcher to interpret the data with the social context of Hong Kong, introducing more than one coder without contextual insight in data analysis may run the risk of destroying the process of interpretation for interpretive inquiry, leading to invalid results and shallow findings (53). Recoding was executed following an initial coding phase of 2 months to reduce inconsistencies in the coded data.

## 3 Results

### 3.1 Participants

Thirty participants aged 20–59 years underwent semi-structured interviews. Their demographic characteristics are provided in Table 1.

**Table 1:** Demographics of the participants.

Informant code	Gender	Age	Education level	Occupation	Monthly personal income (in HKD)	Marital status	Housing	Chronic conditions
P1	F	35	Senior secondary	Full-time education	10,000–19,999	Married	Self-owned public housing	Nil
P2	F	40	Bachelor	Housewife	0	Married	Self-owned public housing	Hypertension, gout
P3	F	39	Bachelor	Housewife	0	Married	Self-owned public housing	SLE
P4	M	30	Senior secondary	Full-time trading	20,000–29,999	Married	Rental private housing	Gout
P5	F	24	Bachelor	Full-time education	20,000–29,999	Single	Rental public housing	Asthma
P6	M	44	Senior secondary	Full-time chef	30,000–39,999	Married	Rental public housing	Hypertension, diabetes
P7	M	38	Master or above	Full-time research	20,000–29,999	Married	Rental private housing	Nil
P8	F	20	Bachelor	Part-time education	10,000–19,999	Single	Rental private housing	Nil
P9	F	32	Bachelor	Full-time education	40,000–49,999	Married	Rental private housing	Nil
P10	M	57	Master or above	Full-time translator	40,000–49,999	Married	Rental private housing	Coronary heart disease, hypertension
P11	F	50	Bachelor	Housewife	0	Married	Rental public housing	Rheumatoid arthritis
P12	F	21	Bachelor	Full-time reporter	10,000–19,999	Single	Rental public housing	Nil
P13	F	29	Master or above	Full-time retailing	30,000–39,999	Single	Self-owned private housing	Nil
P14	F	45	Bachelor	Housewife	0	Married	Rental public housing	Hepatitis B
P15	M	23	Bachelor	Full-time finance and insurance	30,000–39,999	Single	Rental public housing	Nil
P16	F	33	Master or above	Full-time business related	30,000–39,999	Married	Self-owned private housing	Nil
P17	M	28	Bachelor	Full-time accounting	40,000–49,999	Single	Rental public housing	Nil
P18	M	34	Master or above	Full-time transportation	20,000–29,999	Single	Rental private housing	Hypertension
P19	M	24	Bachelor	Full-time research assistant	10,000–19,999	Single	Rental private housing	Nil
P20	M	36	Master or above	Full-time auditing	50,000–59,999	Single	Self-owned private housing	Hypertension
P21	M	48	Master or above	Full-time insurance	40,000–49,999	Single	Self-owned private housing	Gout
P22	M	35	Master or above	Full-time sales and retailing	20,000–29,999	Married	Rental public housing	Nil
P23	M	44	Master or above	Full-time legal consultant	60,000–69,999	Married	Self-owned private housing	Hypertension
P24	M	59	Master or above	Full-time finance	70,000–79,999	Single	Hostel	Hypertension, diabetes, coronary heart disease
P25	M	49	Senior secondary	Full-time bus driver	20,000–29,999	Single	Rental public housing	Chronic renal disease, hypertension
P26	M	50	Bachelor	Full-time finance	80,000–89,999	Married	Self-owned private housing	Hypertension, diabetes
P27	M	46	Bachelor	Full-time sales and retailing	30,000–39,999	Married	Rental private housing	Nil
P28	M	37	Bachelor	Full-time clinic assistant	50,000–59,999	Married	Self-owned private housing	Nil
P29	M	25	Bachelor	Full-time teaching assistant	20,000–29,999	Single	Self-owned public housing	Nil
P30	M	41	Bachelor	Full-time shipping and logistics	50,000–59,999	Single	Rental private housing	Nil

### 3.2 Barriers to vaccination

Although the socio-ecological reasons for vaccine hesitancy have been discussed in the SAGE Vaccine Hesitancy Working Group of the World Health Organization (13) as well as Brewer’s Increasing Vaccination Model (24), the embedded political economy of a vaccine that affects the socio-ecological reasons was not well documented in these existing models. This section will expand the dialogue of these models by systematically investigating the participants’ vaccine refusal in a more comprehensive micro and macro social analysis through the CMA framework with four social levels—individual, micro-social, intermediate-social, and macro-social levels, revealing the intertwining relationships among factors that are relevant to the social-ecological frameworks.

#### 3.2.1 Individual level

The individual level involves barriers related to the participants’ personal experiences with and perceptions of vaccination and the influence of their social network.

##### 3.2.1.1 Lack of trust in vaccines

All participants expressed a low level of trust in COVID-19 vaccines. They doubted the safety of such vaccines because of their recency.

“The vaccine is too new. It was developed in only one year, and I wonder how pharmaceutical companies can research and ensure the safety and reliability of the vaccine in such a short time. I don’t think scientists have enough time to research side effects in such a short time because some side effects may not appear for years. I feel that we are just like white mice, taking part in an experiment for this vaccine.” [P2]

More than half of the participants believed that some side effects of COVID-19 vaccines would not be discovered until after several years. They also suspected that scientists may have concealed the side effects of the vaccine because of commercial concerns. As indicated by this participant, the negative perception of the vaccine is built on the distrust of science, medicine, and medical doctors due to commercial intention.

“I believe that the vaccine definitely has side effects. It is just a matter of time. Of course, those who make the vaccine will not tell you the side effects now because they are selling them. Maybe after 8 or 10 years scientists will tell you the side effects or the vaccine can cause cancer… The scientists invented the vaccine for money. If they want to sell the vaccines, of course they will only tell you the good things and hide all the bad things. Science is not necessarily good, because some people may use science to make more money. You can look at the health maintenance products sold in Watson’s and Mannings [drugstore chains]—these health maintenance products are often connected with scientists and doctors.” [P14]

##### 3.2.1.2 Influence of social networks

The unwillingness of the participants to be vaccinated was reinforced by the opinions of their social network. Lack of trust in COVID-19 vaccines was often prevalent among those in the participants’ social network, discouraging more than half participants from receiving vaccination. The opinions of family members most influenced the participants.

“Most of my family members do not plan to get vaccinated because we are all feeling doubtful about the safety of the vaccine. The vaccine is too new and the data are insufficient. What if the vaccine is proved to have some undesirable outcomes after several years? My family members and I will not want to be a research subject taken part in an experiment.” [P22]

The peers of nearly half the participants also expressed refusal about COVID-19 vaccines, contributing to the participants’ stronger refusal to be vaccinated.

“Most of my friends do not get vaccinated. They were all worried about whether the vaccine is safe because it is so new, and this is my worry, too. Although doctors have talked about the after-effects of the vaccine are mild, who can tell exactly? The exact after-effect really depends on the individual, and I don’t think it can be calculated.” [P13]

##### 3.2.1.3 Worry of burdening family members in case of side effects

Family members were the most prevalent members of the participants’ social networks. Burdening their family members if they experienced long-term side effects from COVID-19 vaccines was a common worry that had prevented almost half of the participants from receiving vaccination.

“If I have any bad complications from the vaccine, my family will suffer because they have to take care of me. My parents are getting old and they are not in very good health, so how can they take care of me? My wife needs to work and take care of two children. If I have any long-term consequences, then she will suffer. I don’t want my family members to suffer.” [P10]

#### 3.2.2 Micro-social level

The micro-social level involves the barriers related to the interactions between healthcare providers and the participants and the interactions among healthcare providers. As demonstrated from the participants, the micro-social level factors of how they perceive the advice from healthcare providers regarding COVID-19 can influence participants’ perceptions and behaviors at the individual level.

##### 3.2.2.1 Medical advice perceived as inconsistent

All participants perceived medical advice about the COVID-19 pandemic as confusing and not trustable because of its inconsistency. Although the medical advice on vaccination was appeared as straightforward to the participants, the inconsistent advice on other preventive measures, such as the use of face masks, had undermined the trust of the participants in biomedicine, and thus in vaccination.

“I feel that the opinions of doctors are always changing. At the very beginning, doctors said it was useless to wear two face masks; they said that wearing two face masks would not give you more protection but could make you more likely to become infected. However, in the fifth wave [of the pandemic], they recommended that you should wear two face masks because the virus is very contagious. How a face mask should be used and how many face mask should be used is scientific knowledge, which should be static. However, their inconsistent opinions make me feel that even science is not trustworthy. Therefore, I feel the same way about vaccination. At this point, they may recommend vaccination, but maybe after months or years, they will say something different.” [P19]

##### 3.2.2.2 Medical advice perceived as lacking consensus

To most participants, biomedicine is a science that emphasizes objectivity, and they expected to receive consistent advice even from different doctors, who should have a consensus. However, almost all participants stated that doctors provided different advice about the pandemic. These diverse opinions not only caused confusion among the participants but also undermined their trust in biomedicine and thus the vaccine.

“At the beginning stage of COVID, doctors suggested that restaurants put partitions between tables to block droplets of the virus and to prevent the spread of infection. However, in the later stage of COVID, another group of doctors suggested that the use of partitions could be harmful because it could impede ventilation and could promote the spread of infection. Then who should I believe? They are all doctors, and I suppose their opinions should be the same, but it turns out that different doctors have different opinions on the same thing, and either side could be wrong. When it comes to vaccination, some doctors would support, but I believe some other doctors may not support.” [P7]

#### 3.2.3 Intermediate-social level

The intermediate-social level involves the relationship and interaction between administration and health care and health policy at an institutional level. As demonstrated from the participants, the intermediate-social level factors were perceived to interact closely with micro-social level factors and can influence participants’ perceptions and behaviors at the individual level.

##### 3.2.3.1 Medical advice perceived as serving institutional interests

Almost all participants perceived that medical advice on vaccination may not be based solely on scientific facts because they expected the vaccination advice provided by doctors to match the government’s vaccination policy. This perception of close relationship between medical doctors and government institutions prevented these participants from trusting COVID-19 vaccines.

“The government is pushing people to get vaccinated. But you know, not everyone listens to the government. The government should know this as well, so the government would work with doctors to promote the vaccine. In this way, I doubt if doctors can really give neutral medical advice about the vaccine because they would not say anything that is against the government’s policy. Doctors’ advice would not be trustable with government’s stepping in.” [P11]

More than half of the participants interpreted the inconsistent advice of medical doctors as supporting changes in the government’s policies of infection control during the pandemic. This perception undermined their trust in medical doctors’ advice on vaccination.

“Doctors work closely with the government to encourage vaccination and other infection control policies. When the government promotes vaccination, doctors would say the vaccine is good. When the government urges people not to eat out, doctors would say that eating out is very dangerous. When the government plans to relax infection control measures, doctors would then give advice that works against what they had proposed before; for example, restaurants do not need to use partitions between tables because partitions prevent air flow. This really makes me lose trust in doctors. Their advice is no longer a neutral medical suggestion, because their standpoints would change to suit government policies.” [P29]

#### 3.2.4 Macro-social level

The macro-social level involves barriers related to the capitalist ideology and the ethnocultural beliefs of medicine and health that are embedded in Chinese culture. As demonstrated from the participants, the macro-social level factors were perceived to interact closely with micro-social level factors and can influence participants’ perceptions and behaviors at the individual level.

##### 3.2.4.1 Vaccine perceived as connected with commercial and capitalist interests

All the participants perceived the COVID-19 vaccine, like other vaccines, is profit-oriented. The participants widely believed that vaccines are invented for money, which undermined their trust in vaccines.

“All vaccines are created for money and profit. If they cannot be sold for profit, do you think the scientists and pharmaceutical companies would still continue to produce vaccines? Obviously, vaccines are not to help people, but to help the capitalists to earn more money. COVID vaccine is even a much bigger business to these capitalists. See how much the vaccine companies have earned.” [P27]

##### 3.2.4.2 Medical advice perceived as connected with commercial and capitalist interests

Almost all participants expressed suspicion about the intentions of medical doctors when recommending vaccination because they perceived that a strong relationship exists between vaccination and the interests of pharmaceutical companies. This perception undermined participants’ trust in COVID-19 vaccines.

“There is a close relationship between healthcare and pharmaceutical companies. They have to rely on each other to survive and to earn more money. Pharmaceutical companies need doctors to buy their products to survive, and doctors need to sell these products to patients to survive. It is all about money and business… A medical school in Hong Kong is working with a pharmaceutical company to develop a new COVID vaccine, and I can expect the doctors in the school would promote the vaccine as a good thing, because they need to sell the vaccines.” [P20]

##### 3.2.4.3 Vaccine perceived as unnatural external intrusion

Almost all participants perceived COVID-19 vaccines as an unnatural intrusion on the body. Many of them felt uncomfortable with the idea of injecting components of a virus into their body.

“I feel the whole idea of vaccination is rather unnatural. I cannot tolerate injecting something chemical and artificial into my body. After all, they are chemicals, and you can never know if these chemicals could be bad for your health. Also, the vaccine is made of virus, and the virus used in the vaccine requires an artificial process. Virus is bad, artificial is bad; I just feel the whole thing is very unnatural, which cannot be good for the body.” [P20]

##### 3.2.4.4 Traditional Chinese medicine perceived as more effective for disease prevention

Nearly half the participants believed that traditional Chinese medicine is more effective in the prevention of COVID-19 than vaccines.

“I think you have to strengthen your body at its core so that you can better protect yourself from infection. You can still get infected after receiving the vaccine if your core is weak. I turn to Chinese medicine to strengthen my core because it has concrete concepts to strengthen immunity. In Western medicine [i.e., biomedicine], doctors only advise sleep more, exercise more, and have a balanced diet. These suggestions are just too general and too commonsense, and you can still get sick even if you take these measures. Chinese medicine has herbal medicine to make your body stronger from the core, and I think this is the most fundamental way to maintain your health.” [P17]

## 4 Discussion

Past studies have reported that a mandatory vaccination policy or vaccination incentive policy is crucial to reach high vaccination coverage (6, 7). However, vaccination incentive policy is not always promising to motivate people to get vaccinated. This study adopted a critical medical anthropology (CMA) framework to investigate why the sampled participants refused COVID-19 vaccination on four social levels, despite the full implementation and enforcement of a vaccination incentive policy Vaccine Pass in Hong Kong. Further to the observation from social-ecological frameworks as suggested by the World Health Organization’s SAGE Vaccine Hesitancy Working Group (13) and Brewer’s Increasing Vaccination Model (24), this study focuses on the context of political economy and how the political economy serves as an underpinning and embedded explanation to the social-ecological model of explanation for the participants’ refusal of vaccination. The four social level analysis according to the CMA framework (26) adopted in this study shows that there is an interacting relationship among the barriers at different social levels, demonstrating that vaccination behavior is a product of a complex social process. The findings of this study revealed that the political economy of Hong Kong society was a crucial underlying factor that explained the participants’ refusal to receive vaccination. Lack of trust in COVID-19 vaccines (individual level) was embedded in the participants’ distrust of biomedicine and medical doctors (micro-social level), and this distrust was rooted in the suspicion of the profit-oriented nature of capitalism (macro-social level) and the political system (intermediate-social level).

The individual level involves barriers related to the participants’ experiences and perceptions as well as the influence of their social network. Most people in the participants’ social networks, such as family members and peers, were also refusing the COVID-19 vaccination. This is consistent with the findings by Sana et al. (54), showing the information from the family was closely associated with one’s vaccination intention against COVID-19. Vaccination was also perceived by the participants as a collective matter that could involve family members if serious side effects arose. The worry about burdening family members—which is also shown to serve as a barrier to receiving COVID-19 vaccination among Chinese older adults (55)—was prevalent among the participants in this study, leading to participants’ vaccination refusal as a result. In addition, safety was a key concern that determined the participants’ vaccination refusal, which is consistent with that of a study conducted in the United States showing that individuals who believed COVID-19 vaccines as unsafe were less willing to be vaccinated (56).

This study further explains that the participants’ perception of vaccine safety was built on their perceived reliability of science, as the participants believed that long-term scientific research is key to establish the safety of COVID-19 vaccines. However, the short history of the COVID-19 vaccine contradicted its safety in the minds of the participants. The participants’ doubts about the safety of COVID-19 vaccines at the individual level were interacting with barriers at the macro-social level, which involves their distrust of capitalism and capitalist institutions that could impede the reliability of science. The participants believed that the safety and reliability of COVID-19 vaccines could only be established without conflict of interest. They expressed strong suspicion toward the COVID-19 vaccine because they perceived there is financial interest of vaccine scientists hidden in its production as conflict of interest. Hall (57) observed people’s skepticism toward health maintenance organizations would increase when medicine and healthcare involve profit-related attributes. This observation is confirmed by another study showing that the profit motives of the vaccine industry would erode people’s trust in vaccination (58). These observations agree with the findings of the present study. The perceived close relationship among vaccination and profit motive of vaccine businesses and producers undermined the trust of the participants. The credibility of medicine and science are undermined when they are connected with commerce and profit motives.

The participants’ lack of trust and confidence in COVID-19 vaccines at the individual level was also intertwined with barriers at the micro-social level involving how the participants interpreted medical advice about vaccination. Their interpretations of medical advice were closely affected by barriers at the macro-social level, again involving their distrust of capitalism. The participants expressed strong suspicion toward the advice of medical doctors who recommended vaccination because they perceived close relationships among medical doctors and profit-oriented vaccine producers and the pharmaceutical industry. The vaccine refusal of the participants, thus, shows a combined force from the interaction between micro-social (i.e., Medical advice) and macro-social (i.e., Capitalism) levels. Confusion regarding COVID-19-related medical advice was another prevalent factor that led to vaccine refusal of the participants. The perceived inconsistent opinions of medical doctors lacking a consensus on certain infection control measures undermined the participants’ trust in medicine and in medical doctors. This inconsistency caused the participants to assume that doctors would vary in their opinions on vaccination, resulting in confusion about whether they should be vaccinated. The participants perceived that the shifting medical advice benefited commercial (macro-social) interests and institutional (intermediate-social) interests. Thus, the participants believed that the vaccination advice provided by medical doctors was not pure medical advice based on science, which undermined their trust in biomedicine and in the advice related to vaccination.

The medical advice related to vaccination at the micro-social level also interacted with institutional vaccination policies at the intermediate-social level, leading to participants’ refusal to the vaccination. The Hong Kong government has widely promoted COVID-19 vaccines through the Vaccine Pass policy. However, the political tension that emerged in Hong Kong before the pandemic had led to a low public trust and high hostility of its citizens toward the Hong Kong Government (59), which ultimately increased the participants’ suspicion and distrust toward the Vaccine Pass. Indeed, trust in government is correlated with people’s response to vaccination policies (60). Government policies that encourage vaccination are not always effective and may generate adverse outcomes, such as arousing public anger and frustration (61–69) and encouraging emigration (63). The participants were feeling skeptical about the medical advice of vaccination because they perceived medical doctors’ support to the vaccination as a reflection of government’s vaccination and other infection control policies. One study asserted that a conflict of interest between medical doctors and policymakers erodes public trust in a vaccine (58). This conflict of interest caused the participants to regard the medical advice related to vaccination as unreliable and suspicious, leading to their vaccine refusal. The political tension emerged before the pandemic had made the medical advice on vaccination to become even more ineffective and incredible to the participants. Albrecht (27) shows that the political distrust and people’s level of satisfaction with governments are strong predictors for Americans’ COVID-19 vaccination acceptance; vaccination rates were found to be much lower in counties where the ruling parties were not in people’s favor.

**Figure 1:**
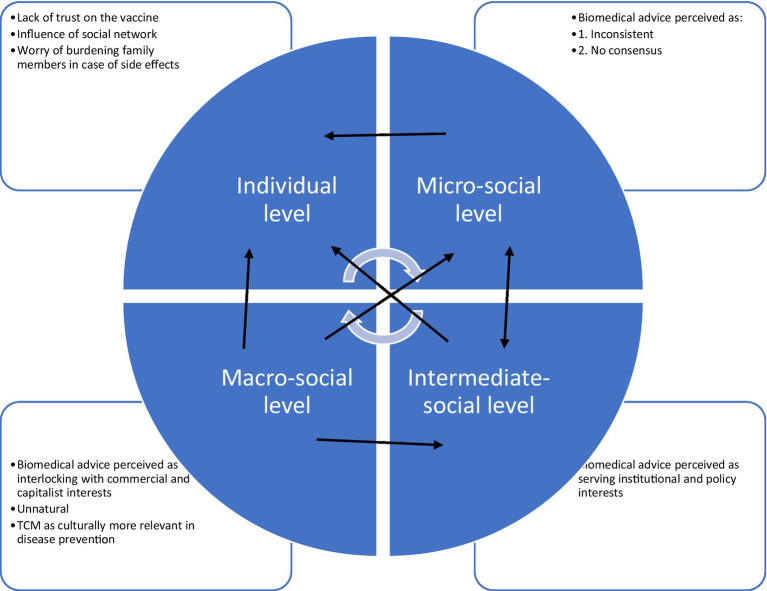
A concept map of the participants’ perceptions for their vaccine refusal.

The results revealed that the refusal of COVID-19 vaccination among the participants involved interactions among factors at the individual, micro-social, intermediate-social, and macro-social levels of the CMA framework (see Figure 1). Fear of COVID-19 is noted as beneficial for containing the pandemic spread as fear can increase people’s risk perception and thus promoting their infection prevention behaviors (70). Although the participants of this study also showed the sense of fear toward the pandemic, it did not promote their vaccination behavior. The participants’ refusal of vaccination was originated in their distrust of medicine and medical doctors at the micro-social level, which was rooted in the profit-oriented principle of capitalism (macro-social level) and conflicting interest with the government’s vaccination policy (intermediate-social level). The participants’ refusal to be vaccinated was not primarily because of their resistance to vaccination as shown from past literature (71, 72); rather, their refusal should be interpreted as a form of resistance against the capitalistic attributes of COVID-19 vaccines and dissatisfaction with the government. Markens et al. (73) argued that considering the rejection of a medical procedure, such as vaccination, as a form of resistance to biomedicine would constitute an oversimplification. People’s decisions about whether to accept or reject a medical procedure are correlated with their concept of “risk” and the attempt to minimize “risk.” For those who reject a medical procedure, the source of risk is associated with the information generated by a procedure that presents a risk (73). Armstrong and Murphy (74) asserted the importance of distinguishing between resistance at the behavioral level (e.g., refusal to accept a particular recommended procedure) and resistance at the conceptual level (e.g., rejection of the discourse within which a particular procedure is embedded). Following Markens et al. (73) and Armstrong and Murphy (74), the “risk” of the COVID-19 vaccine among the participants was rooted in the profit-oriented principle of capitalism (macro-social level) and conflicting interest with the government (intermediate-social level). The participants were not simply resisting the vaccine and the medical advice, but they were resisting the underlying profit-making principle of vaccination and the political pressure to undergo vaccination. To the participants, their fear and risk perception were embedded more in their distrust of vaccine with capitalist implication, biomedicine and the government, rather than the pandemic itself as observed in past literature (70). The adoption of the CMA framework in this study, thus, enabled an investigation of such complicated interaction of different social levels of factors, expanding the understanding of social-ecological model of vaccine refusal.

### 4.1 Limitations

These findings are based on interviews with 30 participants who were anti-vaccinationists of COVID-19 vaccination in the Chinese context at the time of the study. Future studies can recruit more participants from a wider variety of field sites to increase data confidence. Although the findings of this study are based on the contextual analysis in the Chinese context, this article argues that it is important in considering the macro-level political economy as embedded contextual factor in future vaccination intention and barrier research in other communities.

## 5 Conclusion and recommendation to future vaccination policy

Adopting a critical medical anthropology framework, this study investigated the political economy behind the reasons for COVID-19 vaccination refusal, involving intertwining relationships among factors at the individual level, micro-social level, intermediate-social level, and macro-social level. The participants’ lack of trust in COVID-19 vaccines was embedded in the perceived conflict of interest between medical doctors, profit-oriented nature of COVID-19 vaccines, and the government. Following the analysis, enhancing acceptance of COVID-19 vaccination among this subpopulation requires intervention at multiple social levels. First, healthcare and public health institutions are suggested to rebuild the trust between medical doctors and the general public. As the participants commonly perceived medicine and science knowledge as static with a common consensus, multiple medical experts are suggested to provide consistent opinions with a consensus. If changes in their advice have to be made, a clear explanation and justification for making such changes will need to be made clear to the public. Second, in view of the popular distrust on the capitalist implication of the vaccine and on the government among the participants, reaffirmation that vaccination advice is determined solely on the basis of public health and medical concerns without conflicting commercial or governmental interests will need to be made clear to the public. Involving culturally relevant mediators or leveraging community-led vaccination campaigns may also make the vaccination as less suspicious to the anti-vaccinationists.

## Data Availability

The raw data supporting the conclusions of this article will be made available by the authors without undue reservation.

## Appendix A Interview question guide

Probing questions are not indicated in this interview question guide as they were asked in response to participants’ replies.

General questions

1. What is your impression of COVID-19?
2. What is your impression of COVID-19 vaccine?
3. What are the reasons for you not to get vaccinated?

Intermediate-social level

4. The Hong Kong government has implemented the Vaccine Pass since February 2022. Did it serve as a pushing factor for you to consider getting vaccinated? Why/why not?
5. How did you think about the Vaccine Pass policy?
6. How did you think about the Hong Kong government’s response to the pandemic?

Individual level

7. Did you have any family members getting vaccinated? How did your family members view the vaccination?
8. Did you have any friends and peers getting vaccinated? How did your friends and peers view the vaccination?

Micro-social level

9. Have you ever discussed with any healthcare providers about COVID-19 vaccination?
  - If yes, what did you discuss with them, and how did they respond? How did you feel?
  - If no, why did not you discuss with them?
10. How did you think about the healthcare providers’ positions in the vaccination?

Macro-social level

11. Have you ever used any other therapeutic approaches other than western medicine? If yes, what are they? How would you feel about these therapeutic approaches?
12. To you, which therapeutic approach is good to deal with COVID-19 in terms of prevention and treatment? Why?
13. COVID-19 vaccine is free of charge in Hong Kong. How do you think about this vaccine to be offered for free? This free vaccine fails to motivate you to get vaccinated, why?
